# Contraceptives in chronic lymphocytic leukemia (CLL): a narrative review

**DOI:** 10.1007/s00277-026-06830-7

**Published:** 2026-01-17

**Authors:** Rafal Al-Shibly, Nabeel Qassim, Khalil Alfarsi, Salem AlShemmari, Mohammed Yassin

**Affiliations:** 1 Department of Medical Education, Internal Medicine Department, Hamad Medical Corporation, Doha, Qatar; 2 Department of Hematology, National Center for Cancer Care and Research, Hamad Medical Corporation, P.O. Box 3050, Doha, Qatar; 3 Department of Hematology, Sultan Qaboos University Hospital Muscat, P.O. Box 35, Seeb, Oman; 4 Department of Medicine, Faculty of Medicine, Department of Hematology, Kuwait Cancer Control Centre, P.O. Box 42262, Shuwaikh, Kuwait; 5 Department of Medicine, Kuwait University, Kuwait, Kuwait; 6 College of Medicine, Qatar University, Doha, Qatar

**Keywords:** Chronic lymphocytic leukemia, Contraceptives, Targeted therapy, Immunotherapy, Chemotherapy

## Abstract

Women with chronic lymphocytic leukemia (CLL) face unique challenges when it comes to contraception. CLL predominantly affects older adults, with an age-adjusted incidence of approximately 4.7 new cases per 100,000 people per year and a median age at diagnosis of 70 years (Hallek and Al-Sawaf Am J Hematol 96(12):1679–1705, 2021). Although only about 9% of patients are diagnosed before age of 45, those who are of reproductive potential must carefully balance cancer treatment, fertility preservation, and effective contraception. In contemporary practice, most patients are treated with continuous Bruton’s tyrosine kinase (BTK) inhibitors or fixed-duration BCL2 inhibitor–based regimens, while chemoimmunotherapy is reserved for selected cases. These targeted approaches have variable profiles of cytopenias, bleeding and drug–drug interactions and can compromise ovarian reserve and carry teratogenic risks. This necessitates the need for tailored contraceptive counseling within a multidisciplinary oncofertility framework (Oktay et al J Clin Oncol 36(19):1994–2001, 2018).

## Introduction

Chronic lymphocytic leukemia (CLL) is the most common leukemia in adults and predominantly affects older individuals, with an age-adjusted incidence of approximately 4–5 new cases per 100 000 person-years and a median age at diagnosis of around 70 years [1, 2]. Although only a minority of patients are diagnosed before the age of 45, younger women with CLL are increasingly encountered in routine practice due to wider use of laboratory diagnostics.

The therapeutic landscape of CLL has shifted dramatically over the past decade. Chemoimmunotherapy is now rarely used outside selected, biologically favorable subgroups or urgent cytoreduction; instead, first-line treatment for most patients is based on continuous BTK inhibitors or fixed-duration BCL2 inhibitor–based regimens in combination with anti-CD20 antibodies [1]. These agents differ from traditional chemotherapy with respect to gonadotoxicity, bleeding and thrombotic risk, and patterns of cytopenias, all of which may influence contraceptive safety and effectiveness.

For women of reproductive potential, contraceptive counseling must therefore be integrated into contemporary CLL management. The challenge is to balance pregnancy prevention, potential fertility preservation, bleeding and thrombosis risks, and complex drug–drug interactions in the context of long-term oral targeted therapies. This narrative review summarizes available evidence on contraceptive options for women with CLL in the era of BTK and BCL2 inhibitors, and highlights practical considerations for shared decision-making between hematology and gynecology teams.

## Methodology

This narrative review is based on a non-systematic literature search of PubMed, Google Scholar and WHO databases using combinations of the terms ‘chronic lymphocytic leukemia’, ‘CLL’, ‘contraception’, ‘fertility preservation’, ‘BTK inhibitor’, ‘venetoclax’, and ‘targeted therapy’. English-language articles published up to April 2025 were considered, with emphasis on clinical guidelines, randomized trials, large observational studies and expert reviews relevant to contraceptive use in women with CLL. Given the narrative design, no formal inclusion or exclusion criteria or risk-of-bias assessments were applied.

## Contraceptive needs and fertility preservation in women with CLL

Women with chronic lymphocytic leukemia (CLL), particularly those of reproductive age, often require counseling that addresses both fertility preservation and effective contraception. Contemporary CLL therapy is now dominated by targeted agents, although selected patients may still receive potentially gonadotoxic chemoimmunotherapy or radiotherapy, and many agents are teratogenic [1, 3–5]. Consequently, unplanned pregnancy during or shortly after treatment can pose substantial risks to both the mother and fetus. In addition, underlying disease-related immunosuppression and treatment-related cytopenias influence the safety profile of specific contraceptive methods [5, 6].

Ideally, fertility preservation and contraception are discussed together before treatment is initiated. In line with ASCO and other oncology guidance, women who may receive gonadotoxic therapy should be offered referral to reproductive specialists to consider options such as oocyte or embryo cryopreservation or, in selected settings, ovarian tissue preservation [5, 6]. Once a fertility strategy has been chosen—or the patient has declined preservation—contraceptive counseling focuses on preventing pregnancy during treatment and for an appropriate washout period thereafter, while minimizing bleeding, thrombotic and infectious complications and avoiding drug–drug interactions [5–7].

Within this framework, contraceptive choice in women with CLL can be organized into hormonal and non-hormonal methods, each with specific considerations in the context of targeted therapy, cytopenias and immunosuppression [7–9]. See Table 1 for an overview of thrombotic, bleeding and interaction consideration of contraceptive methods in women with CLL.

**Table 1:** Contraceptive methods in women with CLL: thrombotic, bleeding and interaction considerations

Contraceptive method	Thrombosis/bleeding profile	Interactions with BTK & BCL-2 inhibitors	Summary of current evidence and practical considerations
Combined estrogen–progestin oral contraceptives (COCs)	Associated with a 2–3-fold increase in venous thromboembolism (VTE) risk compared with non-use, with higher absolute risk in women with cancer or additional VTE factors [4, 5, 7]. Bleeding pattern usually predictable withdrawal bleeds.	Metabolized via hepatic CYP3A4; clinically important interactions with BTK or BCL-2 inhibitors are uncertain but theoretically possible because of shared metabolic pathways [8, 9].	Generally less suitable for women with CLL who have elevated VTE risk or are receiving targeted oral agents. When contraception is needed, non-estrogenic or non-hormonal methods are usually preferred; COCs may be considered only in carefully selected, low-risk women after individualized assessment [4, 5, 7–9].
Progestin-only pills (“mini-pill”)	Lower VTE risk than combined pills; may cause irregular or unscheduled bleeding but do not appear to increase thrombosis risk [10].	No clear evidence of clinically relevant interactions with BTK or BCL-2 inhibitors; limited data, so routine clinical monitoring is reasonable [8–10].	Often a favorable option for women with CLL who require oral hormonal contraception, particularly when VTE risk is a concern or targeted agents are used. Suitability may be limited by the need for strict daily adherence and by irregular bleeding in some users [8–10].
Levonorgestrel-releasing intrauterine device (LNG-IUD)	Very low systemic hormone levels and not associated with increased VTE risk; frequently reduces menstrual blood loss and may induce amenorrhea over time [10].	No known clinically relevant pharmacokinetic interactions with BTK or BCL-2 inhibitors [10].	Highly effective, reversible method that combines reliable contraception with menstrual suppression, which can be advantageous in women with thrombocytopenia or heavy menstrual bleeding. Insertion should be timed outside periods of severe neutropenia or thrombocytopenia, with attention to infection risk [10, 11].
Copper intrauterine device (Cu-IUD)	Non-hormonal; does not increase VTE risk [11]. May increase menstrual blood loss and dysmenorrhea, which can be problematic in women with baseline menorrhagia or treatment-related thrombocytopenia [11–13].	No pharmacokinetic interactions expected with BTK or BCL-2 inhibitors because the effect is local and non-hormonal [8, 9].	Provides highly effective, long-acting, hormone-free contraception and avoids drug–drug interactions. Use with caution in women at risk of heavy menstrual bleeding; in such cases, an LNG-IUD may be preferable [11–13].
Progestin implant	Very low-dose progestin with low VTE risk, similar to other progestin-only methods; may cause irregular or infrequent bleeding and, in some users, amenorrhea [11].	Metabolized via hepatic enzymes, but no robust evidence of clinically significant interactions with BTK or BCL-2 inhibitors; theoretical CYP3A4 effects have not clearly compromised efficacy [12, 13].	Long-acting reversible option that avoids estrogen and has minimal interaction concerns. Suitable for women who prefer a “forgettable” method and do not wish to use an intrauterine device; counseling regarding the possibility of irregular bleeding is important [11–13].
Barrier methods (condoms, diaphragm, cervical cap)	No systemic hormonal exposure and therefore no additional VTE risk; do not alter menstrual bleeding patterns [14].	No interactions expected with BTK or BCL-2 inhibitors [14].	Safe, hormone-free methods that also provide protection against sexually transmitted infections when condoms are used. Because typical-use effectiveness is lower than that of intrauterine or implantable methods, they are best used as additional protection or when pregnancy risk is acceptable [11, 14].
Female sterilization (tubal ligation/salpingectomy)	No ongoing effect on VTE risk after recovery from surgery; brief perioperative risk related to anesthesia, immobility and any procedure-related bleeding [14, 15].	No drug–drug interactions with BTK or BCL-2 inhibitors; perioperative timing should take into account cytopenias and bleeding risk if the patient is on active therapy [14, 15].	Permanent option appropriate only for women who are certain they do not desire future pregnancy. Requires careful preoperative assessment in CLL to minimise surgical and bleeding risks and should be discussed in the context of overall prognosis and life plans [11, 14, 15].

### Hormonal contraceptives: mechanisms and considerations

Hormonal contraceptives include combined estrogen–progestin formulations (oral pills, patches and vaginal rings) and progestin-only methods (pills, injections, implants and levonorgestrel-releasing intrauterine systems). These methods primarily act by suppressing ovulation, thickening cervical mucus and altering the endometrium [8, 9].

In the general population, combined hormonal contraceptives are highly effective; however, in women with CLL their use warrants caution. Estrogen-containing methods increase baseline venous thromboembolism (VTE) risk and this risk is further amplified in women with cancer, coexisting cardiovascular risk factors or concomitant antithrombotic therapy [7, 10–14]. In addition, estrogen-containing contraceptives may interact pharmacokinetically with targeted therapies metabolized via CYP3A4, including BTK and BCL2 inhibitors [3, 15–17]. For these reasons, combined hormonal methods are often less suitable in this population, especially in those treated with BTK or BCL2 inhibitors or with significant VTE risk factors [7, 10–14].

Progestin-only methods generally have a more favorable hemostatic profile and are therefore attractive options [7, 13, 14]. Oral progestin-only pills, subdermal implants and levonorgestrel-releasing intrauterine systems provide highly effective contraception without added estrogen-related thrombotic risk and can also induce partial or complete menstrual suppression, which is beneficial in women prone to heavy menstrual bleeding during thrombocytopenia [7, 13, 14, 18]. Injectable progestins offer long-acting contraception, but potential metabolic effects (e.g. on weight and bone density) and the need for scheduled injections should be considered in patients with limited mobility or frequent hospital visits. For all hormonal methods, possible drug–drug interactions and the patient’s overall thrombotic and bleeding risk should be reviewed, ideally in collaboration with gynecology [5–7].

### Non-hormonal contraceptives: alternatives for women with CLL

Non-hormonal contraceptive methods are an important option for women with CLL who prefer to avoid hormones or for whom hormonal methods are contraindicated. These approaches avoid systemic hormonal exposure and pharmacokinetic interactions with targeted therapies, while still providing effective pregnancy prevention when used correctly [7–9].

The copper intrauterine device (IUD) is the most effective non-hormonal method. It works by releasing copper ions into the uterine cavity, creating a local inflammatory environment that is toxic to sperm and oocytes and impairs fertilization [8, 9]. Because it does not rely on systemic hormones, the copper IUD does not increase thrombotic risk and has no known direct interactions with BTK or BCL2 inhibitors [7, 14]. This makes it an attractive choice for many women with CLL who require long-acting, highly effective contraception. However, copper IUDs can increase menstrual blood loss and dysmenorrhea; in patients with baseline menorrhagia, treatment-related thrombocytopenia or a history of heavy menstrual bleeding, this potential for increased bleeding must be weighed carefully against the benefits [7, 14, 18]. In addition, women with profound or prolonged neutropenia may have a higher risk of pelvic infection, so careful screening, counseling and follow-up are essential [5–7].

Barrier methods—including male and female condoms, diaphragms and cervical caps—provide contraception by physically preventing sperm from reaching the cervix. They are generally safe in women with CLL, do not interact with systemic therapies and, in the case of condoms, also reduce the risk of sexually transmitted infections [7–9]. Their main limitation is lower typical-use effectiveness compared with intrauterine or implantable methods, as they depend on correct and consistent use at every act of intercourse. For many women with CLL, barrier methods are best used as an adjunct to another highly effective method, such as an IUD, rather than as the sole form of contraception when pregnancy would pose substantial risk [7, 9, 14].

For women who have completed their families or who are certain they do not desire future pregnancy, permanent sterilization can be considered. Laparoscopic tubal ligation or other sterilization procedures offer highly effective, irreversible contraception [19]. In the context of CLL, the decision must account for perioperative risks, including bleeding and infection, particularly in patients with cytopenias or significant comorbidities. Thorough counseling, involving both hematology and gynecology when possible, is crucial to ensure that permanent contraception aligns with the patient’s long-term health goals and anticipated disease course [5–7].

## Special considerations for women on targeted therapy and immunotherapy

Targeted agents and monoclonal antibodies now form the backbone of CLL therapy, while conventional chemoimmunotherapy is increasingly reserved for selected clinical scenarios [1, 3, 4]. For women of reproductive potential, these regimens raise specific issues related to embryo–fetal toxicity, bleeding and cytopenias, and potential drug–drug interactions with hormonal contraceptives. Contraceptive counseling should therefore be closely integrated with treatment planning and adapted to the characteristics of each therapeutic class [5–7]. See Table 2 for an overview of hemostatic and pharmacologic considerations of CLL therapies in relation to contraception.

**Table 2:** Hemostatic and pharmacologic considerations of CLL therapies in relation to contraception

Drug class/examples	Hemostatic/cytopenia profile	Fertility & pregnancy	Contraception considerations (summary of evidence)
BTK inhibitors (ibrutinib, acalabrutinib, zanubrutinib)	Common mucocutaneous and procedure-related bleeding due to qualitative platelet dysfunction; mild–moderate thrombocytopenia; venous thrombosis appears less frequent and usually occurs in the presence of additional cardiovascular or treatment-related risk factors [12, 16, 17, 20, 21].	No clear evidence of human gonadotoxicity; preclinical data suggest embryo–fetal toxicity. Pregnancy is contraindicated during therapy and for a short post-treatment interval as recommended in product information and guidelines [1, 20, 21].	Non-estrogenic or non-hormonal methods are generally favoured (progestin-only pills, implant, LNG-IUS; copper IUD with caution in women with heavy menses). Combined estrogen methods are often avoided in women with significant VTE or cardiovascular risk or concomitant antithrombotic therapy. Ongoing monitoring for heavy menstrual bleeding is important during treatment [3–5, 7, 10, 11, 14, 20, 21].
BCL2 inhibitor–based fixed-duration regimens (venetoclax + obinutuzumab/rituximab)	Neutropenia and thrombocytopenia are frequent, especially during ramp-up and early cycles; bleeding is mainly related to thrombocytopenia rather than qualitative platelet defects; no clear intrinsic prothrombotic signal has emerged from clinical trials [13, 18, 21].	Embryo–fetal toxicity has been observed in animal and human data; pregnancy is contraindicated during treatment, and effective contraception is advised during therapy and for ≥ 30 days after the last dose according to product labeling [18, 19, 21, 22]. Long-term fertility data are limited.	Time-limited regimens allow a defined duration of contraception. Long-acting non-estrogenic methods that provide menstrual suppression (LNG-IUS, progestin implant) may be helpful in women at risk of heavy bleeding during thrombocytopenia. Copper IUDs offer a hormone-free option but may exacerbate menorrhagia and should be used cautiously in patients with baseline or anticipated thrombocytopenia [10, 11, 14, 18, 19, 21, 22].
Chemoimmunotherapy (FCR, BR; now used in selected patients)	Transient but sometimes profound cytopenias (neutropenia, thrombocytopenia); bleeding risk is primarily driven by thrombocytopenia and supportive therapies; underlying malignancy confers an elevated baseline VTE risk [1, 20, 21].	Alkylating agents such as cyclophosphamide are potentially gonadotoxic, and many cytotoxic agents are clearly teratogenic. Pregnancy is contraindicated during treatment and for several months afterwards, and ovarian reserve may be reduced [1, 6, 20, 21].	Fertility preservation should be discussed before starting therapy in women who may desire future pregnancy. Highly effective contraception is strongly recommended during and after treatment. Intrauterine methods are best inserted outside periods of severe neutropenia or thrombocytopenia; LNG-IUS can additionally help control heavy menstrual bleeding in women with treatment-related cytopenias [3, 6, 10, 11, 20, 21].
Anti-CD20 monoclonal antibodies and other immunotherapies (rituximab, obinutuzumab)	Infusion reactions and transient cytopenias are common; repeated courses can cause hypogammaglobulinemia and increased infection risk; no consistent strong intrinsic prothrombotic signal [1, 20, 21].	Not clearly gonadotoxic, but embryo–fetal risk and transient neonatal B-cell depletion have been reported. Contraception is advised during therapy and for an extended post-treatment interval (months) according to individual product labels and guidelines [1, 20, 21].	Reliable contraception is needed throughout treatment and the recommended washout period. Methods should be chosen with attention to infection risk and cytopenias; intrauterine devices are generally acceptable with appropriate timing of insertion and follow-up. Barrier methods, particularly condoms, remain important for sexually transmitted infection prevention and as back-up to other methods [3, 11, 14, 20, 21].

### BTK inhibitors (continuous therapy)

Bruton’s tyrosine kinase (BTK) inhibitors, such as ibrutinib and acalabrutinib, are widely used as continuous oral therapies in CLL [1, 3, 4]. Their most characteristic hemostatic toxicity is bleeding, ranging from low-grade mucocutaneous bleeding to major hemorrhage, largely driven by qualitative platelet dysfunction [17, 20, 22]. Thrombotic events are reported but appear less common and are often associated with additional cardiovascular risk factors or concomitant antithrombotic medications [17, 20, 22]. When counseling women receiving BTK inhibitors, the primary concerns are therefore bleeding risk, management of heavy menstrual bleeding and interactions with antiplatelet or anticoagulant therapy, rather than thrombosis alone [7, 18, 20, 22].

Non-estrogenic methods that do not further increase bleeding or thrombosis risk and are not strongly dependent on CYP3A4 metabolism—such as levonorgestrel-releasing intrauterine systems, progestin-only pills and implants, or non-hormonal methods—are generally favored [7, 13–15]. Combined estrogen–progestin contraceptives are often avoided in women with significant cardiovascular or VTE risk factors or those receiving concomitant antithrombotic therapy [7, 10–14]. Product information and major guidelines also recommend effective contraception during BTK-inhibitor treatment and for at least one month after the last dose because of potential embryo–fetal toxicity [3, 4].

### BCL2 inhibitor–based fixed-duration regimens

Venetoclax-based fixed-duration regimens, most commonly venetoclax plus obinutuzumab or rituximab, are now standard options in frontline and relapsed CLL [1, 3, 4, 21]. This time-limited approach has distinct contraceptive implications compared with continuous BTK-inhibitor therapy. Women typically require highly effective contraception for the duration of venetoclax treatment and for an additional washout period (at least 30 days after the last dose, according to product labeling, because of potential embryo–fetal toxicity) [3, 21]. Thereafter, pregnancy may be considered if disease status, organ function and overall prognosis permit, ideally in consultation with hematology and reproductive specialists [5, 6].

Cytopenias, particularly neutropenia and thrombocytopenia, are common during venetoclax dose escalation and combination therapy [3, 21]. Heavy menstrual bleeding during periods of thrombocytopenia is a frequent practical problem and may exacerbate anemia or necessitate unscheduled dose interruptions [7, 18]. In this setting, long-acting non-estrogenic methods that can suppress menstruation, such as levonorgestrel-releasing IUDs or progestin implants, may improve tolerability, provided individual thrombosis risk and potential drug–drug interactions are considered [7, 13, 14, 18]. Copper IUDs remain an attractive non-hormonal option with minimal interaction potential, but their tendency to increase menstrual blood loss must be weighed carefully in women at risk of thrombocytopenia or baseline menorrhagia [7, 14, 18]. Close coordination between hematology and gynecology teams is essential to manage heavy menstrual bleeding while maintaining adherence to the fixed-duration regimen [5–18] See Table 2.

### Anti-CD20 monoclonal antibodies and other immunotherapies

Anti-CD20 monoclonal antibodies such as rituximab and obinutuzumab are integral components of both chemoimmunotherapy and targeted combinations [1, 3, 4]. These agents can cause profound but usually reversible B-cell depletion and are associated with embryo–fetal risk; women of reproductive potential are advised to use effective contraception during therapy and for 6–18 months after the last dose, depending on the specific product label [3, 4]. Transient B-cell depletion has been reported in infants exposed in utero, which has implications for neonatal vaccination schedules [3].

For women receiving anti-CD20 antibodies with or without BTK or BCL2 inhibitors, contraceptive choice should take into account infection risk, cytopenias and infusion schedules [3, 5–7]. Highly effective long-acting methods (intrauterine devices, implants, or sterilization where appropriate) reduce the need for frequent clinic visits solely for contraception and minimize the risk of unplanned pregnancy during periods of immunosuppression [7, 9, 19]. Barrier methods, particularly condoms, remain important as adjunctive methods for sexually transmitted infection prevention [7, 9].

Overall, in women with CLL receiving targeted therapy or immunotherapy, contraceptive counseling should emphasize the need for reliable contraception for the full period of embryo–fetal risk defined in drug labels, careful management of bleeding and cytopenias (including heavy menstrual bleeding), and avoidance of estrogen-containing methods in those with significant thrombotic or cardiovascular risk [3–20, 22]. These principles are intended as a synthesis of current evidence and product information to support shared decision-making, rather than as formal clinical guidelines.

## Discussion

Chronic lymphocytic leukemia (CLL) primarily affects older adults, but a minority of patients are women of reproductive age who require careful counseling on contraception, fertility and treatment-related risks. In the era of targeted therapy, contraceptive decision-making must consider not only baseline venous thromboembolism (VTE) risk, but also bleeding complications, cytopenias, potential teratogenicity and drug–drug interactions [1, 3, 6, 23]. This narrative review synthesizes available evidence to outline key issues relevant to contraceptive choice in women with CLL receiving contemporary therapies.

BTK inhibitors, such as ibrutinib, have transformed CLL management and are commonly used as continuous oral therapies [1, 3, 23]. Their predominant hemostatic toxicity is bleeding, ranging from low-grade mucocutaneous bleeding to more serious hemorrhage, largely mediated by qualitative platelet dysfunction; thrombotic events are reported but appear less frequent and often occur in the presence of additional cardiovascular risk factors or concomitant antithrombotic agents [17, 20, 22]. Because BTK inhibitors are metabolized through CYP3A4, co-administration with estrogen-containing contraceptives may theoretically alter hormone exposure, although robust clinical data are limited [15]. In practice, non-estrogenic methods—including progestin-only pills, implants, and levonorgestrel-releasing intrauterine systems—and non-hormonal options are generally favored, particularly in women with elevated VTE risk or concurrent antiplatelet or anticoagulant therapy [3, 8–14, 23]. Attention to heavy menstrual bleeding (HMB), which may be exacerbated by platelet dysfunction or thrombocytopenia, is essential when selecting and monitoring contraceptive methods, and may warrant closer collaboration with gynecology [6].

Venetoclax-based fixed-duration regimens have become standard of care in both frontline and relapsed CLL, most commonly venetoclax combined with obinutuzumab or rituximab for a predefined treatment period (e.g. 12 or 24 months) [1, 3, 21, 23, 24]. This time-limited approach has important implications for contraception and reproductive planning. In contrast to continuous BTK-inhibitor therapy, women receiving fixed-duration venetoclax-based regimens may require highly effective contraception only for a defined interval covering treatment and an appropriate washout period, after which pregnancy may be considered if disease status and organ function permit, in line with product labeling and guideline recommendations [3, 24–26]. Cytopenias, particularly neutropenia and thrombocytopenia, are common during venetoclax dose escalation and combination therapy [3, 21, 24]. In women who menstruate, HMB during periods of thrombocytopenia can be clinically significant and may necessitate temporary treatment interruption, iron supplementation or gynecologic intervention [3, 6]. Non-estrogenic methods that provide endometrial suppression—such as levonorgestrel-releasing IUDs, progestin-only pills or implants—can reduce menstrual blood loss and may improve tolerability of targeted therapy in women prone to HMB [3, 9, 13, 14]. In contrast, copper IUDs may increase menstrual flow and dysmenorrhea in some users; this potential trade-off should be discussed carefully in patients with baseline menorrhagia or anticipated thrombocytopenia [9, 14]. Multidisciplinary collaboration with gynecology can facilitate timely interventions, including short-term tranexamic acid or hormonal strategies, while minimizing disruptions to CLL therapy [3, 6].

Immunotherapy, particularly anti-CD20 monoclonal antibodies such as rituximab, is frequently combined with chemoimmunotherapy or targeted agents [1, 3, 23]. These drugs are associated with transient cytopenias, hypogammaglobulinemia and increased infection risk, and available data suggest embryo–fetal toxicity and transient neonatal B-cell depletion in exposed pregnancies; contraception is therefore recommended during treatment and for a prolonged post-treatment interval as advised in product information and clinical practice guidelines [3, 23]. Invasive methods such as IUD insertion should be timed to avoid periods of profound neutropenia or thrombocytopenia, but intrauterine contraception can generally be used safely with appropriate screening and follow-up [8, 9, 14].

Conventional chemoimmunotherapy regimens, such as fludarabine–cyclophosphamide–rituximab or bendamustine–rituximab, are now reserved for selected patients but remain relevant in some settings [1, 3, 23]. Alkylating agents may be gonadotoxic and are clearly teratogenic; as a result, fertility preservation discussions should occur before starting treatment in women who might desire future pregnancy, and reliable contraception is essential during therapy and for a defined interval thereafter [6]. Cytopenias and hepatotoxicity may further influence contraceptive safety and timing of invasive procedures [6, 8, 14].

Available data and existing guidance suggest several broad principles when selecting contraception for women with CLL. International family-planning guidance, including the WHO Medical Eligibility Criteria, and oncology-focused contraceptive reviews describe non-hormonal methods (e.g. copper IUDs and barrier methods) as highly effective, free of systemic hormone exposure and not subject to drug–drug interactions; they are generally acceptable in women receiving cytotoxic or targeted therapies, provided there is no severe neutropenia or active pelvic infection [8, 9, 14]. Progestin-only methods (progestin-only pills, implants and levonorgestrel-releasing IUDs) also have a low thrombosis risk and minimal interaction with targeted agents, and may be particularly useful when menstrual suppression is desired, especially in patients at risk of HMB and thrombocytopenia [3, 9, 13, 14]. In contrast, estrogen-containing contraceptives carry a higher baseline risk for venous thromboembolism and potential CYP3A4-mediated interactions, so they are generally less appropriate in women receiving BTK or BCL-2 inhibitors or in those with additional VTE risk factors [3, 10–15, 17, 20]. Regardless of the selected method, monitoring hematologic parameters—including platelet counts and hemoglobin—is important when bleeding patterns may be altered, and treatment plans should be revisited if HMB, anemia or thrombotic complications occur [3, 6, 8, 14].

These principles are not intended as formal clinical guidelines but as a synthesis of current evidence and expert opinion to support individualized, shared decision-making between patients, hematologists and gynecologists. Aligning contraceptive choice with contemporary CLL treatment strategies, bleeding and thrombosis risks, and patients’ reproductive goals can help optimize safety while respecting patient preferences and quality of life [1, 3, 6, 23].

This review has several limitations that should be acknowledged. First, it is a narrative rather than a systematic review, and we did not perform a formal quality assessment or meta-analysis of the included studies; the selection and interpretation of evidence may therefore be subject to publication and selection bias. Second, much of the available literature on contraception in cancer is derived from mixed oncology populations or general gynecologic guidance rather than CLL-specific cohorts, so several of our conclusions rely on extrapolation from broader cancer or immunocompromised populations rather than prospective data in women with CLL. Third, data on the reproductive safety, drug–drug interactions and menstrual outcomes of contemporary targeted agents—particularly BTK and BCL2 inhibitors—remain limited, and product-label recommendations continue to evolve. Consequently, some of the proposed principles are based on expert opinion and current guideline statements rather than high-level comparative evidence. Finally, this review does not capture patient-reported outcomes or preferences, which are critical for truly shared decision-making in contraceptive care. These limitations highlight the need for prospective studies and CLL-specific registries addressing fertility, contraceptive use and menstrual health outcomes in women receiving modern CLL therapies.

## Conclusion

Contraception in women with chronic lymphocytic leukemia (CLL) is a multifaceted aspect of care that extends beyond simple pregnancy prevention. Decisions must balance treatment-related teratogenicity, bleeding and thrombotic risk, cytopenias, potential drug–drug interactions and the woman’s own fertility goals. In contemporary practice, most patients are treated with BTK inhibitors or fixed-duration venetoclax-based regimens, with chemoimmunotherapy and anti-CD20 antibodies used in selected settings. This therapeutic shift places particular emphasis on managing heavy menstrual bleeding in the context of thrombocytopenia and platelet dysfunction, and on matching contraceptive duration to whether therapy is continuous or time limited.

Across available options, non-hormonal methods and progestin-only long-acting reversible contraception emerge as particularly useful tools. Copper IUDs and barrier methods avoid systemic hormones and drug–drug interactions, while levonorgestrel-releasing IUDs and progestin implants add the benefit of menstrual suppression for women at risk of heavy bleeding. Estrogen-containing contraceptives remain an option only for carefully selected, lower-risk patients because of their higher baseline venous thromboembolism risk and potential pharmacokinetic interactions with targeted agents. Fertility preservation discussions—where gonadotoxic therapy is planned—should occur before treatment initiation, and ongoing contraceptive counseling should be revisited as disease status, therapy and reproductive plans evolve. These principles, grounded in current evidence and expert guidance but not intended as formal clinical recommendations, highlight the need for individualized, multidisciplinary care involving hematology, gynecology and, when appropriate, fertility specialists to ensure that contraceptive strategies are safe, acceptable and aligned with each woman’s priorities.

## Data Availability

All data and information regarding this clinical review is available as part of the article and no additional source data are required.

## Declarations

### Use of AI-assisted tools

ChatGPT (OpenAI) was used for language and style editing under author supervision. The authors reviewed all AI-assisted text and remain responsible for the final content.
